# Remarks upon the State of Medical Science, and Some of the Diseases of Syria and Arabia

**Published:** 1857-09

**Authors:** R. Gutzlaff Barclay


ART. I.—*Remarks upon the State of Medical Science, and Some of the Diseases of Syria and Arabia.* By R. GUTZLAFF BARCLAY, M. D.

PRESUMING that a few reflections upon the present condition of medical science, in the land of the learned Alkhendi, the illustrious Rhazes, Avicenna, the Prince of Physicians, and Moses, the great lawgiver, may be deemed neither inapposite nor uninteresting, the following pages are devoted to that subject, and respectfully presented in lieu of a more formal treatise upon some more hackneyed theme.

In order that a just comparison may be instituted between the diseases and medical practice current at the period alluded to, and those now obtaining, a brief notice of the varied state of the climate, necessarily claims attention.

The subject upon which the following remarks are submitted, has been under constant investigation during several years' residence and practice in Jerusalem and its vicinity, a point nearly midway in position between the extremes of the region under consideration, and possessing, within the space of a few hours' travel, every variety of climate found between Antioch, on the one side, and the Persian Gulf and Red Sea, on the other; the precise area embraced in the writings of the authors above alluded to.

In forming a correct estimate of the diseases generally prevalent in this region, compared with those existing in similar latitudes in America, it should be borne in mind, that the isothermal line is much higher in the Old, than in the New World; consequently, although this region lies nearly between the same parallels of latitude as those embraced between Washington City and New Orleans, the climate is materially warmer; and, in contrasting the present catalogue of diseases with that of the period referred to (once prevalent in this same territory), it must be remembered, that, in consequence of diminished amount of rain, rivers, then figuring upon its map, now no longer exist.

Elevation of temperature, diminution of humidity and verdure (and the consequent electrical changes), sparsity and deterioration of food and drink, are all the necessary sequents of this long partial suspension of the "latter rains."

It must, however, by way of offset to these and other circumstances tending to aggravate or multiply diseases, also be mentioned, that the very lavish use of ablutions, enjoined by the arch-impostor of Mecca (whose followers constitute the chief class of inhabitants of all this region of country), exerts no little influence in mitigating, and even averting, many diseases. But, notwithstanding these modifying circumstances, and numerous other chemical and physical changes of a most signal character (to say nothing of the political and social changes, tending to exercise an alterative influence upon disease and its treatment, to which this land and its population have, for the last score or two of centuries, been subjected), we nevertheless find the diseases to be substantially the same as they are (incidentally, 'tis true) reported to have been, in the writings of Moses, that oldest of all historians; and in the subsequent work of "Ahrun, Priest of Alexandria."

The appended abstract—an average of several years' observation—will fully show the present thermometric, barometric, and pluviometric state of the climate of the central portions of the region under consideration. The hygrometric index almost uniformly points to one extreme in winter, and to the other throughout the summer; indicating saturation with moisture in the former, and the almost total absence of humidity during the latter season.*

It may be remarked, in general, however, of the climate, both of the mountainous and champaign portions, that it is excessively arid in summer, and moist in winter. During the winter, easterly winds prevail; but in the summer, the wind blows from the northwest with the regularity of a sea breeze; continuing, without interruption, daily, from ten A.M. till a late hour at night.

Warm, dry and oppressive siroccos occasionally blow from the region of the sandy deserts of the south, for several days successively, at all seasons; and when these alternate with the chilling blasts from Lebanon's ever snow-capped summit, the vicissitudes are both sudden and very great. It is a subject of general remark, that the winds blow in spells of several days' continuance; and those from the west and southwest (which prevail from November to April), are, by the Arabs, called "the fathers of the rains."

While snow very rarely falls at Jerusalem (and generally dissolves immediately), dews are much more copious than in similar occidental climes.

There are a few hoar frosts, of quite a formidable appearance, but they seem scarcely destructive of the tenderest vegetable life; while ice, even in the thinnest pellicle, is a phenomenon of very rare occurrence. The winters are so mild that the orange, lemon, date, fig, apricot, pomegranate, almond, and other delicate trees, shrubbery, and plants, require no protection whatever.

* It may be here mentioned, in connection with this fact, that, during the greatest atmospheric aridity, in midsummer, the very minutely ramifying fibres of the roots of a certain plant, having the power of penetrating the smallest crevices of the limestone rocks, may be observed with numberless drops of water pendent from them in certain cases; and this phenomenon is particularly observable in a recently exhumed sepulchre on the brow of the valley "Ben Hinnom," in which are several large mounds of the debris of human skeletons, thrown pell-mell together in masses.

It is a favorite saying of the Arabian poets, that "Sannin"—a spur of the Lebanon range—"bears winter on his head, spring on his shoulders, and autumn in his bosom; while summer lies sleeping at his feet."

The country around about Jerusalem combines a rare diversity of climate; the temperature of the Dead Sea and plain of the Jordan, some fifteen miles distant, very closely resembling that of Egypt and Lower Arabia, the upper districts being very cool, and the city itself presenting a fair average of the entire region.

To the Arabian or Saracenic physicians, who first rendered chemistry (however magistral the science was in the hands of the theorizing Alchemists) practically subservient to medical science, are we indebted for many valuable medicines and chemical agents and appliances. The untiring researches of the Alchemists after an "elixir vitæ," whereby to prolong human existence indefinitely, the philosopher's stone, for the transmutation of the baser metals into gold—however chimerical—resulted in wrenching from nature's grasp, some of the most valuable agents and materials to be found in the laboratory of the Pharmaceutist; as alcohol, opium, squill, rhubarb, senna, scammony, the golden henbane, thornapple, elaterium, the mandrake, &c. &c. That the peculiar toxical properties of this latter root may be the better understood, it may not be deemed entirely irrelevant to give a brief outline of its effects, as displayed in the person of a Mr. L——, of Scotland, who while on a visit to the Holy City, had the curiosity to test its edible qualities. After being boiled some ten hours, he observed that the root became no softer, though the water was made as black as ink; it was therefore sliced and fried in butter, and served up at tea. On account of the exceeding bitter and unpalatable taste it still retained, he was able to eat only a few small slices; enough, however, not only to turn his teeth and lips black, but produce very curious effects on his entire system. He soon yielded to the comatose powers of the narcotic root, and retired to rest; passing a very unpleasant night, troubled with startling dreams and frightful visions. On arising the next morning, he fancied there was a company of street musicians performing in his room, very much to his annoyance; and still more to his discomfiture, he imagined a number of serpents to be around him, in killing which, he utterly demolished not only his fragile crockery ware, but the more substantial furniture of his room; and was so busily engaged defending himself, that his toilette was not completed till about four P.M. Every string or straw, in his wild hallucinations, assumed the terrific form of a serpent, and was most carefully dropped in alcohol for preservation. On being discovered, the pupillary window of his eyes was dilated almost to the entire obliteration of the iris; pulse very hard, incompressible, and frequent (being 140 per minute), and he talked incessantly and incoherently about the serious losses he had sustained by the fancied robbers with whom he had been battling for many hours.

Being a man of full habit, he was blooded freely, dry-cupped along the spine, and copiously purged by the croton oil. But it was several days before he became materially convalescent, and the dilatation of the pupils continued for many days.

As a treatise of this character would be imperfect without some mention of the mode of living amongst the Arabs (who constitute the main body of the inhabitants of this country), it may be allowable to briefly enumerate some of the most ordinary articles of food and drink; as well as their manner of dress, and such habits as may have a bearing upon the subject under consideration. While the beard is allowed to grow untrimmed in all its native luxuriance, the head is closely shaven every week,* as well as all other parts covered with hair, except that portion of the scalp over the occipital tubercle, where a small tuft is left for the firmer grasp of the Resurrection Angel; which is to elevate him to the Mahomedan Paradise. Their heads are protected by the seamless red Fez (Tarboush) alone, or more generally by a muslin shawl wrapped tightly around this cap, constituting the picturesque-looking turban.

The female suffers hair to grow abundantly on her head, but with religious scrupulousness removes it from all other parts of the body, either by plucking it out with the hand, or preferably by means of a paste composed of an article called "dibbiz," made from the grape, and then inspissated to the thickness of pitch, which when applied warm, and allowed to cool, effectually extracts every hair when forcibly jerked away.

The feet of the lower classes, though generally unshod, are sometimes protected by sandals; and the middle order wear a rough shoe, made of untanned leather; while the upper circle uses a yellow morocco stocking in an outer loose red or yellow slipper, made of goat or sheep skin. The only garment worn by the great majority of persons throughout all this region of country, during the summer months, consists of simply a loose cotton gown, extending to the knees, and secured "about the loins with a leathern girdle," or a long narrow strip of cotton cloth; and during the severest months of winter, the only addition made to this very primitive costume, is a loose woollen robe; constituting their dress by day and bed-clothing at night, which must be totally inadequate to the demands of nature, as they sleep upon a thin piece of matting spread over a dirt or plastered floor.

The fruits ordinarily raised are figs, pomegranates, oranges, lemons, the prickly-pear, apricots, pears, almonds, olives, dates, bananas, and grapes of admirable quality, which are in season from the middle of July to November,

* This tonsillary operation is sometimes performed with a depilatory mixture of freshly slaked lime, and other materials.

and are very abundantly converted into wine by the Jews and Christians, and into raisins by the Mahomedans. Among the vegetables grown here, may be enumerated as the principal crops, lentils, onions, egg-plants, squash, cucumbers, melons, and cauliflowers—all of the finest quality. Leaven bread is rarely eaten: the only kind in use being that made of wheat, very coarsely ground (or rather bruised) in a hand-mill, by the women at the dawn of day, and baked in a very small low mud-oven (heated by the refuse of the camel), into exceedingly thin leathery cakes, called "taboon bread." A species of dirty, red, gravelly rice is very plentifully consumed when but partially cooked with "semny," the fat extracted from the broad adipose caudal appendage of the sheep of this country, or with "serage," the oil expressed from the seed of the "susim" plant.

Coffee is used on all occasions, at all hours of the day, and very strong, but in small quantities and without sugar.

After shaking milk an hour or two in a goatskin, suspended from a rude tripod made by tying together three inclined poles, a very rich and delicious drink results from the partial separation of the butter; but their "lebban," or naturally soured milk, is by no means entitled to the praise so lavishly bestowed upon it by thirsty travellers, when "hardly bestead and hungry."

For several months raw cucumbers (rind and all, without the least condiment), constitute almost the only article of food for the lower class; no wine, ale, porter, cider, or intoxicating potation of any kind is used by the Moslem population—lemonade and "sherbet" (made of sugar, rose-water, and lemon juice), being their only beverages. "Arak," a highly rectified alcoholic drink, and wine, are, however, coming into very general favor with the Jews and Christians, and are beginning also to be used clandestinely, but to an alarming extent, among the higher classes of Mahomedans.

Domestic fowls thrive well, with very little attention, and are very prolific; wild partridges (in this country equalling a hen in size) are quite numerous, and herds of beautiful gazelles—the "hinds and roes" of Scripture—roam freely over the hills and valleys; but in consequence of the great nudity of the country, wild game generally is scarce.

Their meats consist almost exclusively of mutton and goat flesh—the prejudice of the Mussulmans and Jews against swine being so strong that they deem it a religious duty to pelt them with stones whenever they meet the unfortunate creatures in passing the well-supplied convents of the Christians (where alone they are to be found); but camel's flesh, which is equally prohibited by the Pentateuch, is considered a rare delicacy by the Turks as well as Christians. Beef is an article of great scarcity, and especially so in the cities, being strictly embargoed at the gates, by the laws of the land, on account of the practice, so universally current among the peasants, not to butcher their cattle till hopelessly sick or ready to die from accidental injury. Snails are abundantly consumed by all, but crabs only by the Frank portion of the population, being a commodity so scarce in market that the lower classes cannot afford to purchase them. Olive berries, either salted or pickled, as well as the raw oil expressed from them, are used in great abundance.

Although tobacco is so strictly proscribed by their "great Prophet and Lawgiver," yet they are so excessively addicted to smoking (both men, women, and children), that a pipe, with flint, steel, and spunk, and a well-supplied scrip, forms an indispensable "*vade illiscum*," even while taking the shortest walk, as well as during their frequent cessations from labor. Let them, however, have the full benefit of the fact, that they are not at all guilty of the practice of chewing, and but very slightly addicted to that scarcely less odious habit of snuffing "the noxious weed."

Opium is used for inebriating purposes by their moolahs or priests, both in substance and in smoke.

The following list includes those diseases of most frequent occurrence in Jerusalem, and (as far as can be ascertained) throughout this region of country generally, which, however much it may differ from accounts given by Eastern travellers, may be relied on with entire confidence.

Ophthalmic diseases generally—inclusive of conjunctivitis (its most frequent form),—iritis not unfrequent, and cataract.

Fevers—intermittent, remittent, continued, and "Syrian fever."

Hypertrophy of spleen, as consequent upon intermittent fever.

Dropsies and hernias.

Cutaneous affections generally.

Tape-worm, and other verminous affections.

Hepatitis, both acute and chronic.

Mania à potu, that most horrifying disease with which nearly every occidental part of the globe is cursed, is almost entirely unknown throughout the Orient. But so far as ascertainable, none of the diseases mentioned in the writings of the Arabian physicians, or in the Sacred Record (excepting those of a supernatural character), have disappeared—unless, indeed, leprosy may possibly present an exception to the remark: while diseases of a modern origin are perhaps as rife here, as in any other part of the Orient. That ophthalmic affections should be so prevalent, is not at all astonishing, when we consider the direct exposure to light and heat, to which the eye is constantly subjected throughout a large portion of this country, and the mechanical injury sustained from the dust and fine sand carried about by the winds of the desert districts; but especially the manner in which the infants of the peasantry are always carried about, suspended in a coarse bag from their mother's backs with their faces turned upwards, their eyes fully exposed to the bright effulgence of a Syrian sun; nor does the tarboush or turban afford any material protection in after-life.

The poor diet mainly used, and the crowded state in which the greater portion of the inhabitants of Syria and Arabia live, dwelling in caves and but very imperfectly ventilated huts, are *alone* sufficient to account for the universal prevalency of intermittent, remittent, and continued fevers. The "Syrian fever" is well known as the most malignant form of continued fever, of a decidedly typhoid tendency, to an attack of which, every foreigner almost without exception is subject, while undergoing the acclimating ordeal; but the universal prevalence of the intermittent (or "burning ague," as styled by Moses), is a matter of such marvel, that many resolve it into a fulfilment of the judgment denounced against the land for disobedience.

But when we take into consideration the mass of filth and debris of every kind found in all oriental cities, amounting in Jerusalem to some forty feet in depth, as was actually ascertained in excavating a foundation for the Prusso-Anglican Church on Mount Zion, and other buildings; the heaps of decomposing animal matter (for Jerusalem is a perfect internecropolis, the dead being commingled with the living, and in some places lying above them), and remember that they derive their supplies of water chiefly from tanks filled by the rains, and teeming with animalcules and filth of every description—no great surprise need be felt at the extensive prevalence of every variety of intermittent. So strong is the tendency to intermittents, that many diseases of quite an anomalous character either resolve themselves into well-developed intermittents in a short time, or readily yield when treated by quinia even when contraindicated by the pulse. While upon the subject of fevers, it may not be out of place to remark, that "Henna" (a dye-stuff in popular use for tattooing the hands), is only applied to *the feet* during the existence of fever, it being supposed to be possessed of refrigerant properties; also "kohl," another dye-stuff of a blue color, is universally applied to the eyelids of new-born babes, for forty days after birth—sanitary ophthalmic properties being ascribed to it.

For the relief of obstinate bilious affections, copious draughts of human urine are prescribed; and as a sheet-anchor of trust for difficult intermittents, the inhalation of the fumes arising from scorpions thrown on burning coals of fire, is strongly recommended.

In proof of the extraordinary love of this people for physic, I will give the following extract from a book entitled "Three Weeks in Palestine." After alluding to the fact that no person is allowed to practise the therapeutic art at Constantinople without a license from government, the writer says, "A man had set up a doctor shop without this diploma; the police were sent to apprehend him, but instead of seizing the culprit, they allowed him to slip quietly away, while they made a rush at his phials and gallipots, and swallowed indiscriminately the whole contents of his physic-shop. Luckily it consisted of simples only, and no harm was done."

Hypertrophy of the spleen as a consequence of the very imperfect manner in which the native doctors treat intermittents, is constantly met with; but though intermittents prevail so extensively, yet they are not at all obstinate in their character, and very readily yield to the simplest medication. The writer has frequently prescribed for not less than fifty cases per day, simply a purgative, to be followed by several days' use of Fowler's arsenical solution, with uniform success. Dr. Mundlesohn of the French hospital here established, has written an essay, the object of which is to prove that the remarkable prevalence of intermittents in Jerusalem, is due to absence of salts in the water of house-tanks, the only kind used in Jerusalem; but on trial, the water when well filtered is found to have a sp. gr. of 1002½, and hence must hold saline matter in solution, as indeed might very properly be inferred, from the fact that it necessarily passes over surfaces plastered with mortar made of lime and ashes.

Cutaneous affections are quite as prevalent here as in the United States; and after attaining an advanced stage, they become the favorite nidus for the deposition of larvæ of various kinds; hence they are of a most repulsive, and often obstinate character.

Syphilitic affections are not so prevalent as might be expected, while gonorrhœal discharges are quite common. Some of these disorders (strange and disgraceful as it may appear) are ascribable to sexual intercourse with animals. For not only is masturbation or self-pollution rife here, but that abominable crime, so strongly reprobated by the Apostle Paul in the 27th verse of the first chapter of Romans, and also those prohibited under so severe a penalty by the great Jewish lawgiver (Lev. 20 : 15–16), are unblushingly practised on female goats and donkeys; not only by reckless youth, but by married men, whose harems are plentifully supplied with even a liberal Mussulman allowance of wives!

Dropsies (as might very reasonably be expected of a dense population, so poorly fed and clothed) are quite prevalent, but promptly yield to medical treatment. As a cause of the frequency of hernial protrusions, the almost universal habit of wearing a stiff leathern girdle very tightly drawn around the waist, may be assigned as not at all impossible.

Palpitation of the heart is sometimes met with, and rheumatism is by no means as rare as might be supposed in so genial a climate; while hepatic complaints occur quite as frequently as might be expected of much hotter latitudes. For rheumatic pains in the legs, the patient is severely flogged several times a day over the shoulders. Small-pox, a disease mentioned as early as the year A. D. 622, by Ahrun, Priest at Alexandria, in his work entitled "*Pandects,*" as well as plague and cholera, though originating and for a long while confined to this part of the globe, certainly are by no means so rife as in former years. This is attributed by many to the cleanly influence exerted by "Franks," who for the last score or two of years, have been settling in the tents of Shem, in rapidly increasing numbers.

Certainly the abatement of these scourges of the human family, is not attributable to the trivial though rigid, preventive measures practised at the various quarantine stations; nor can their introduction from abroad be prevented by the very puerile sulphurous fumigation to which letters and imported articles have heretofore so vigorously been subjected. Indeed, his highness the Sultan has become so fully convinced of the utter futility of these quarantine sanative measures, that he has recently ordered their abandonment, except during the actual existence of plague and other contagious diseases.

The following is a brief account of the treatment of small-pox, as practised by the native physicians of this country. Nothing is done for a week, but on the seventh day, a gargle of *white* lamb's urine is ordered, and if there be any eruption on or about the eyes, they are to be washed with the urine. Then from the seventh day, a strong gargle of aloes is daily used till the forty days are accomplished. Though the small-pox raged so furiously in Jerusalem, decimating its population, yet it failed to extend to Bethlehem, only some seven miles distant. The Turks, and indeed all Mussulmans are such stanch fatalists, that, although the efficacy of vaccination is so well known to them, yet it was not until the present Sultan Abd-El-Mejid encouraged its adoption by very handsome "*bucksheeshes*," that it has been much resorted to, even at Constantinople itself!

Some idea may be had of the present state and standing of the medical faculty of this country, by the following extracts from the autobiography of an educated Arab physician, of my acquaintance, of Yaffa, or Jaffa (the ancient Joppa). He says, "The medical staff, in this country, is not the brilliant one of the Arabian age;—that has passed by. It may now be divided into four classes: first, those who belong to it by descent; and, together with the honor inherited from their fathers, remnants of Arabic manuscripts, recipes from old, and from the works of Avicenna, they make a livelihood; give cooling drinks, made from certain herbs; feel the pulse; carry smelling-bottles under their belts; and a stick with a silver top, and look very dignified.

"The second class are the masters of families, who give barley-water for fever, broken bones, dislocations, and every complaint.

"The third class are the barbers, who are dashing practitioners, bleed in every case, put on plasters, never operate, and let the poor patient struggle on, under the bleeding system.

"The fourth class are those who cure by charms and superstition. For example, if the poor man has the ague, they order him to be laid near the tomb of some saint belonging to his creed.

"H. R. H. Prince Wali told me, that a Tartar, who was very fond of eating, stuffed himself till he was seized with a terrible colic. The doctors who were sent for, belonging to this latter class, brought two bags of holy earth from the tomb of a certain saint, and crammed them into the mouth of the unfortunate victim, till he was nearly stifled. In this state, the Prince saw him, and inquired what was the matter. "Your Royal Highness," they replied, "he is so stuffed, we cannot get the medicine down." One of them proposed to push it down with a ramrod. The Prince drove away the holy doctors with their remedy, and gave the Tartar a dose of medicine, which relieved him.

"Sometimes they bring holy stones, holy bones, and holy water, and place them at the tombs; and when they are on the brink of the grave, direct them to some old rug." (Dr. Kyat.)

In Jerusalem, the Moolahs or Priests (who also serve as medical men) are the favored and distinguished class; for physicians here are held in the highest esteem.

No such thing as a medical school is known throughout all Syria and Arabia; hence, the practice of medicine is altogether empirical and unavailing.*

There are many cases of great longevity to be met with here; due, no doubt, to the mild and equable climate, a good constitution, abundant exercise in the open air, and great simplicity of diet.

Superstition being the natural offspring of ignorance, the people of every caste and condition, throughout this extensive and highly favored region of the globe (where ignorance sways its leaden sceptre from the rude Bedouin of the desert to the lordly Turk), are all exceedingly superstitious. The mere touch of a crazy man is believed to be effectual for the cure of all manner of diseases; and such persons are held in high respect and esteem, if not *veneration*, by all. Amulets of precious stones, gold cases containing choice sentences from the Koran, &c., are almost universally worn; and written prescriptions are very frequently *eaten*, as well as *smoked*, as a curative measure.

Incantations and tractions—not very unlike those once practised in Great Britain—are frequently resorted to by the higher order of physicians.

Necromancy and witchcraft were probably scarcely more rife in this land, even when the great lawgiver Moses enacted such severe prohibitions against

* Although the present enterprising Sultan established a handsomely endowed Medical College at Constantinople, and, by way of encouragement, even gave a premium for students, and ventured so far to outrage the feelings of his subjects as to order dissections to be made, the experiment proved an entire failure.

those practices, than are *pretensions* to such powers at the present day. Owing to their superstitious regard for the sacred month of "Ramadan," it is considered a misdemeanor, of no low grade, to take medicine during that entire moon, although the excesses of which they are guilty, in the debaucheries and feastings indulged in throughout every night of "Ramadan" (when they fast and sleep during the day, and feast and frolic all night), render its administration of the first importance.

The use of cathartics is well understood and appreciated; enemas are also frequently resorted to: but emetics appear to be a remedy in not very high favor with any class of people in this region. Diaphoretics are ordered, however, for almost every affection, while the warm bath, being in such universal use for mere comfort and cleanliness, is never prescribed therapeutically.

Salivation is a very favorite remedy, though rather indiscriminately put in requisition sometimes: it is never produced by absorption through the skin or stomach, but always through the lungs, by inhaling the vapor of an article that appears to be native red cinnabar, thrown on burning coals. For the cure of ptyalism the Arab doctors give a weak solution of tobacco, in which powdered alum is dissolved. There exists so marked a tendency to salivation among this people, that calomel in ordinary doses, even when combined with a purgative more active than itself, will not unfrequently bring on profuse salivation, despite our most vigilant counteraction.

Bloodletting is in high repute; and not only does general venesection receive its full share of attention, but also the topical abstraction of blood. In cupping, they exhaust the atmosphere by means of lighted conical slips of paper, and scarify with a razor, by making several deep gashes transversely over the temporal regions, and others in a vertical direction.

The following outline of the treatment of femoral bruise, which the writer witnessed not long since in Bethlehem, may suffice to impart some idea of their limited knowledge of surgery. The first application consisted of a plaster of incense and arrak; the latter appeared to be alcohol of low sp. gr., and the incense, such as is used for thurifying the altars of Oriental churches, chiefly of a terebinthine character.

A purge was next given, and the contused part washed in vinegar; a piece of recently slaughtered sheepskin was now suffered to remain on for one day, and then the fatty oil of the tail of the sheep, oil of peppermint, and a peculiar kind of black seed, boiled together and used as a liniment. On the following morning a thick slice of the warm muscle of a freshly slain sheep, sprinkled with sugar, was applied for one hour, for the purpose of determining the exact seat of the injury; and when thus ascertained, the doctor ordered the ramrod of his pistol to be heated to redness, which he then applied in several places, over the seat of greatest pain, till the integuments were fried to a crisp, ashes and oil, however, having previously been rubbed on to designate the place of application of the cautery.

In answer to my query as to the average frequency of the pulse per minute, in a normal condition of the system, the learned doctor (who was also a chief of distinction from a neighboring village), very confidently replied, "About four beats per minute!!" It may be supposed that he is as poor a doctor as surgeon, judging from one of his remarks, that "fire was the best medicine he possessed!" Oriental physicians of the present day, unlike the learned Alkhendi of ancient times, never regulate their doses, and explain their *modus operandi*, by geometrical propositions and musical harmony.

The Arabs use the actual, as well as the potential cautery, on almost all occasions, both upon man and beast. Albucasis, "the bold and dexterous surgeon," could not have been more unnecessarily severe and lavish in the use of the knife and hot iron, than are his degenerate successors. Abundant evidence of this is had in their method of treating mania; which, whatever be its cause, nature, or stage, is committed to the sheikh or chief of the tribe, who confines the unfortunate victim closely in irons in some cheerless dungeon, and there restricting him to "bread of affliction, and water of affliction," administers *flagellations ad libitum*, even to groans and blood; and it is indeed astonishing to note how frequently they recover.

The natives of this country are entirely unacquainted with the use of the catheter; but have so far advanced as to make a rude application of the splint. Of minor surgery, circumcision is the *major* operation; in the performance of which, constant practice has rendered them quite expert. This important chirurgico-ecclesiastical operation being one of such great import, is intrusted alone to the highest order of medical functionaries, in order to secure its performance, *secundem artem—à la Koran.*

Since the introduction of sulphur matches into that part of the globe, a specific for itch is had by rubbing up their ends with olive oil.

It appears that the Turkish, Jewish, and Arab women of the present day, are as "lively," as were the Hebrew females of yore in Egypt; and on account of their exemption from such artificial causes as render parturition difficult and dangerous in more refined circles, they have no occasion for obstetrical interference; hence professional accoucheurs are quite unknown. A different inference however might probably be drawn, from the extremely tender age at which marriages are contracted! It is a common custom for boys to marry at the age of twelve or fourteen, and girls as early as ten or eleven, or even younger!

It is not a usual thing for the female attendant (should there be one) to make an examination "per vaginam," or render the slightest assistance in the delivery of the child and after-birth. Peasant women are delivered while sitting on a step, stone, or some other low seat; being merely held sometimes by an attendant, while another squats in front with a cloth spread ready to catch the infant as it is protruded out! For tardy delivery of the placenta, the patient is ordered to blow forcibly through her hands; or in the event of this failing, to immerse her feet in cold water. Great virtue is ascribed to the little blue-bead, worn during utero-gestation, as a preventive of the afterpains.

Abundant use is made of *heralding women*, for the purpose of announcing to the neighborhood, in shrill pæans which all must hear, the joyful fact, that a Mussul*man* is born into the world; and the first informant of the happy father is entitled to a present, rating from fifty cents upwards, according to the rank and liberality of the overjoyed father.

Having already far exceeded the limit contemplated in commencing this hasty treatise, the writer will not farther extend it, except merely in conclusion, to briefly contrast and compare a certain hideous disease, now existing in particular localities in Palestine, with that described in the Sacred Writings as leprosy.

It is a much mooted point, whether the leprosy now to be met with in Jerusalem and other portions of the East, is identical in character with that described by Moses; but from the investigation bestowed upon the subject, the writer does not hesitate to affirm that it is quite a different affection. It is strongly contradistinguished, by the entire exemption of clothing and the walls of the houses. It is true there is a vulgar prejudice against inhabiting a house on the inner walls of which green splotches appear, and these certainly are unhealthy; for they are always so damp when thus discolored as to afford a suitable nidus for the growth of cryptogamous plants, and naturally predispose to disease, but never to leprous affections. The present leprosy is not contagious, infectious, or congenital; and does not always itch. The progeny of lepers are not necessarily leprous, if one parent only be affected; indeed both parents may be leprous, and yet their progeny escape. It sometimes suppurates, but never so closely resembles a furuncle as to be confounded with it. There is also an entire absence of the bright white spots. It appears to be deeper-seated than the leprosy of the Bible, and the extremities—withering and suppurating—throw out spiculum after spiculum and bone after bone, till reduced to a mere stump a few inches in length.

### THERMOMETRICAL, BAROMETRICAL, AND PLUVIOMETRICAL OBSERVATIONS, MADE AT JERUSALEM, PALESTINE, DURING THE YEAR 1854.

The thermometer used in these observations is one of McAllister's best instruments, though not self-registering. It was kept suspended at a short distance from the wall, in a small isolated stone building, which, though well ventilated during the day, was usually closed at night, and on this account the sunrise temperature should be rated a degree or two lower perhaps in winter than the recorded numbers. The temperature was carefully noted thrice a day,—sunrise, noon, and sunset.

It should also be remarked, that the barometer made use of had been rather roughly handled, but while its indications for the ascertainment of the actual height of the atmospheric column may have been thus vitiated, its relative indications, which alone affect the design of this table, are perfectly reliable.

Jerusalem (though there are some discrepancies in regard to its *exact* elevation), it is well ascertained, is situated rather less than three thousand feet above the level of the Mediterranean Sea, and more than four thousand above that of the Dead Sea, whose singular depression, about one thousand feet below the Mediterranean and Red Seas, is a well-ascertained fact.

According to information obtained through the Foreign Office, from the Admiralty in London, Jerusalem is situated in

Latitude N. 31° 46′ 35″,

Longitude E. 35° 18′ 30″ from Greenwich.

*Table of Average Monthly Temperatures.*

| YEARS. | January. | February. | March. | April. | May. | June. | July. | August. | Sept. | October. | Nov. | Dec. |
|---|---|---|---|---|---|---|---|---|---|---|---|---|
| | ° | ° | ° | ° | ° | ° | ° | ° | ° | ° | ° | ° |
| 1851, . . | | | | | | 72·8 | 79·8 | 78·2 | 75· | 72·3 | 67· | 53·3 |
| 1852, . . | 49·6 | 52·1 | 56· | 62·2 | 69·6 | 73·8 | 78· | 78· | 74·1 | 76·6 | 62·7 | 55·3 |
| 1853, . . | 51·4 | 60·4 | 60·2 | 64· | 77·6 | 77·3 | 78· | 80· | 80·2 | 74·9 | 61·1 | 52·9 |
| 1854, . . | 49·6 | 50·8 | 51· | 58·1 | 74·1 | 76·9 | 80·8 | 80·9 | 77·3 | 72·9 | 64·3 | 56·6 |
| 1855, . . | 47·1 | | | | | | | | | | | |
| Average, . | 49·4 | 54·4 | 55·7 | 61·4 | 73·8 | 75·2 | 79·1 | 79·3 | 77· | 74·2 | 63·8 | 54·5 |

Average annual temperature, 66·5°.

*Register of the Fall of Rain at Jerusalem from* 1846 *to* 1854 (*in inches*).

| MONTHS. | 1846–7. | 1847–8. | 1848–9. | 1849–50. | 1850–1. | 1851–2. | 1852–3. | 1853–4. | Average |
|---|---|---|---|---|---|---|---|---|---|
| October, . . . | 4· | 4· | 0· | Not registered. | 0· | 0· | 0· | ·2 | 1· |
| November, . . | 6·4 | 0· | ·2 | | 6·4 | 0· | 1·8 | 2·3 | 2· |
| December, . . | 0· | 19· | 16· | | 33·8 | 15·2 | 9·4 | 3·6 | 14· |
| January, . . . | 9·8 | 24·6 | 19·4 | | 14·6 | 13·6 | 4·2 | 4·4 | 13· |
| February, . . | 32·8 | 5·8 | 13·2 | | 24· | 25· | 4· | 5·8 | 16· |
| March, . . . | 6· | 0· | 11·8 | | 4· | 8·8 | 21·4 | 6·5 | 8· |
| April, . . . . | 0· | ·2 | 0· | | 2·2 | 0· | 1·2 | 3·6 | 1· |
| May, . . . . | 0· | 1·4 | 0· | | 0· | 24· | 2· | ·5 | 1· |
| Total per annum, | 59· | 55· | 60·6 | | 85· | 65· | 44· | 26·9 | 56·5 |

